# Do No Harm: Efficacy of a Single Herbicide Application to Control an Invasive Shrub While Minimizing Collateral Damage to Native Species

**DOI:** 10.3390/plants8100426

**Published:** 2019-10-18

**Authors:** David J. Gibson, Lindsay A. Shupert, Xian Liu

**Affiliations:** 1 School of Biological Sciences, Southern Illinois University Carbondale, Carbondale, IL 62901, USA; lindsayshup@siu.edu (L.A.S.); xianliu2014@siu.edu (X.L.)

**Keywords:** Community composition, Exotic species, Grassland, Herbicides, *Lespedeza cuneata*, Phylogenetic diversity, Restoration, Seed additions

## Abstract

Control of invasive exotic species in restorations without compromising the native plant community is a challenge. Efficacy of exotic species control needs to consider collateral effects on the associated plant community. We asked (1) if short-term control of a dominant exotic invasive, *Lespedeza cuneata* in grassland restorations allows establishment of a more diverse native plant community, and (2) if control of the exotic and supplemental seed addition allows establishment of native species. A manipulative experiment tested the effects of herbicide treatments (five triclopyr and fluroxypyr formulations plus an untreated control) and seed addition (and unseeded control) on taxonomic and phylogenetic diversity, and community composition of restored grasslands in three sites over three years. We assessed response of *L. cuneata* through stem density counts, and response of the plant community through estimates of canopy cover. Herbicide treatments reduced the abundance of the exotic in the first field season leading to a less dispersed community composition compared with untreated controls, with the exotic regaining dominance by the third year. Supplemental seed addition did not provide extra resistance of the native community to reinvasion of the exotic. The communities were phylogenetically over-dispersed, but there was a short-term shift to lower phylogenetic diversity in response to herbicides consistent with a decrease in biotic filtering. Native plant communities in these grassland restorations were resilient to short-term reduction in abundance of a dominant invasive even though it was insufficient to provide an establishment window for native species establishment.

## 1. Introduction

Control of invasive exotic species in grassland restorations offers many challenges [1,2]. There is the primary need to slow or halt the spread of invasive species, especially those that may become dominant in a restoration. However, analogous to the Hippocratic oath for physicians, there is also the need to “do no harm” to the native species community in what is often a challenging setting. As with decision making in the release of genetically modified organisms (GMOs) and environmental decision making [3,4], the precautionary principle would caution against the use of broad-spectrum herbicides, although they may be justified when invasive species assume dominance.

To address the first challenge of controlling an invasive species, appropriate control methods need to be used that are both logistically and economically appropriate over what may be large areas of invasive species infestation. While methods of manual control such as hand cutting or pulling may be desirable, more feasible chemical controls using herbicides are often applied [5,6]. In restorations, there is the risk that non-target species may be negatively affected by herbicides [7]. Closely related desirable species may be inadvertently affected through collateral damage even when using selective herbicides for treating an invasive. Appropriate herbicides therefore have to be carefully chosen based upon composition of the background native species community. 

At each stage of a restoration there will be dominant species interacting with a suite of subordinate species. The interactions between a dominant exotic and subordinate native species may be quite different to those between a native dominant species and its co-occurring subordinate species [8,9]. Thus, removal or control of a dominant invasive exotic species in a restoration can affect the desirable subordinate species in an unanticipated manner. As a dominant exotic is reduced in abundance, biotic filters may be relaxed as niche space becomes available. In addition, if appropriate control of an exotic is obtained, supplemental seed additions of native species may drive establishment of a diverse native community resistant to reinvasion of the exotic [10,11]. Such seed additions can offer an opportunity to increase the abundance of desirable species [12]. 

Recognition of the changes in community assembly mechanisms and niche relationships in managed restorations can be facilitated through measurements of phylogenetic diversity in addition to conventional measurements of taxonomic diversity [13] if interpreted with caution [14]. As a measure of biodiversity, phylogenetic diversity differs from taxonomic diversity as it takes into account the phylogenetic and evolutionary relationships among taxa based upon distance matrices of pairwise dissimilarities derived from molecular data [15]. Taxonomic and phylogenetic diversity patterns may differ over the course of a restoration reflecting key and heretofore unknown aspects of community assembly [13,16,17].

In this study, we tested the efficacy of herbicide control on a highly invasive herbaceous perennial, *Lespedeza cuneata* (see Section 4.1 Study Species), on the subordinate species in grassland restorations. Following label guidelines using recommended herbicides, we monitored effects on the target invasive and the subordinate species over three subsequent years. Because *L. cuneata* is such an aggressive perennial that produces a large quantity of viable seed, and can resprout unless the roots are killed [18], we predicted that herbicide use would provide short-term control of *L. cuneata* but that there would be collateral damage to native species. We also predicted that short-term control of the invasive would allow successful establishment of desirable species introduced by seeding.

## 2. Results

### 2.1. Site Characteristics

Across all sites, 75 plant taxa were identified (Table S1). Over 4 years of this experiment, *Lespedeza cuneata* was the most abundant species with a mean cover of 38.0% ± 1.4%. Other abundant species over this time period included *Solidago canadensis* (17.3% ± 1.0%), *Sorghastrum nutans* (14.9% ± 1.1%), *Andropogon gerardii* (9.6% ± 1.0%), and *Bromus racemosus* (7.0% ± 0.8%); all other species had a mean cover of less than 5%.

### 2.2. Treatment Effects on Lespedeza Cuneata

At the start of the experiment, abundance of *L. cuneata* at the 3 sites was high (Stem density Site 1 = 69.6 ± 6.4 stems per m^2^, Site 2 = 71.5 ± 8.1 stems per m^2^, Site 3 = 97.5 ± 8.0 stems per m^2^; Cover Site 1 = 31.2% ± 3.4%, Site 2 = 38.8% ± 4.4%, Site 3 46.2% ± 3.3%). Treatment effects are presented for only stem density as stem density and cover of *L. cuneata* were highly correlated across all sites (Pearson correlation coefficient R = 0.75, n = 809, *p* < 0.0001) and gave qualitatively similar results. 

**Figure 1:**
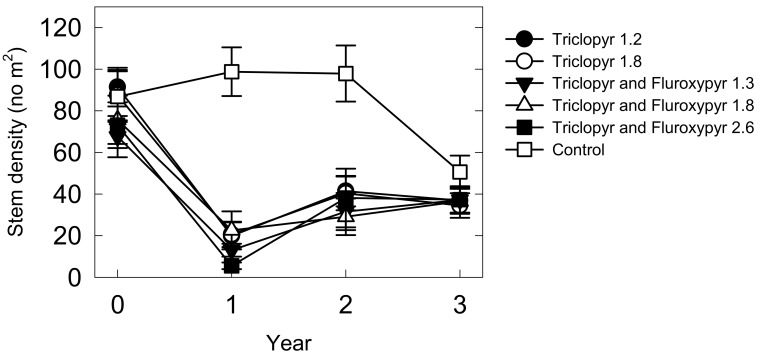
Stem density ± standard error bars of *Lespedeza cuneata* over three years following herbicide treatments (year zero) or in untreated control plots across three prairie restoration sites. Stem density was significantly higher (*p* < 0.05) in control versus herbicide treatments (see Table 3 for details) in years 1 and 2 but not in years 0 or 3. In year 1, stem density in triclopyr 1.2, triclopyr 1.8, and triclopyr and fluroxypyr 1.8 herbicide treatments were significantly higher than stem density in the triclopyr and fluroxypyr 2.6 herbicide treatment.

There was a significant interaction between herbicide treatment and time on stem density across all sites (F_15,358_ = 6.40, *p* < 0.0001). There were no main effects of seed treatment, or interactions with year and/or herbicide treatment on stem density (*p* > 0.05 in all cases). There were no differences between treatments in year 0, but compared with the untreated control plots, all the herbicide treatments reduced stem density of *L. cuneata* by 70%–96% across all sites in years 1 and 2 following treatment (Figure 1). In year 1 the herbicide treatment H5 (triclopyr and fluroxpyr 2.6) reduced stem density more than treatments H1, H2, and H4 (triclopyr alone or with fluroxpyr 1.8). By the third year there were no differences in stem density among herbicide treatments and untreated control plots. 

### 2.3. Taxonomic and Phylogenetic Diversity

**Figure 2:**
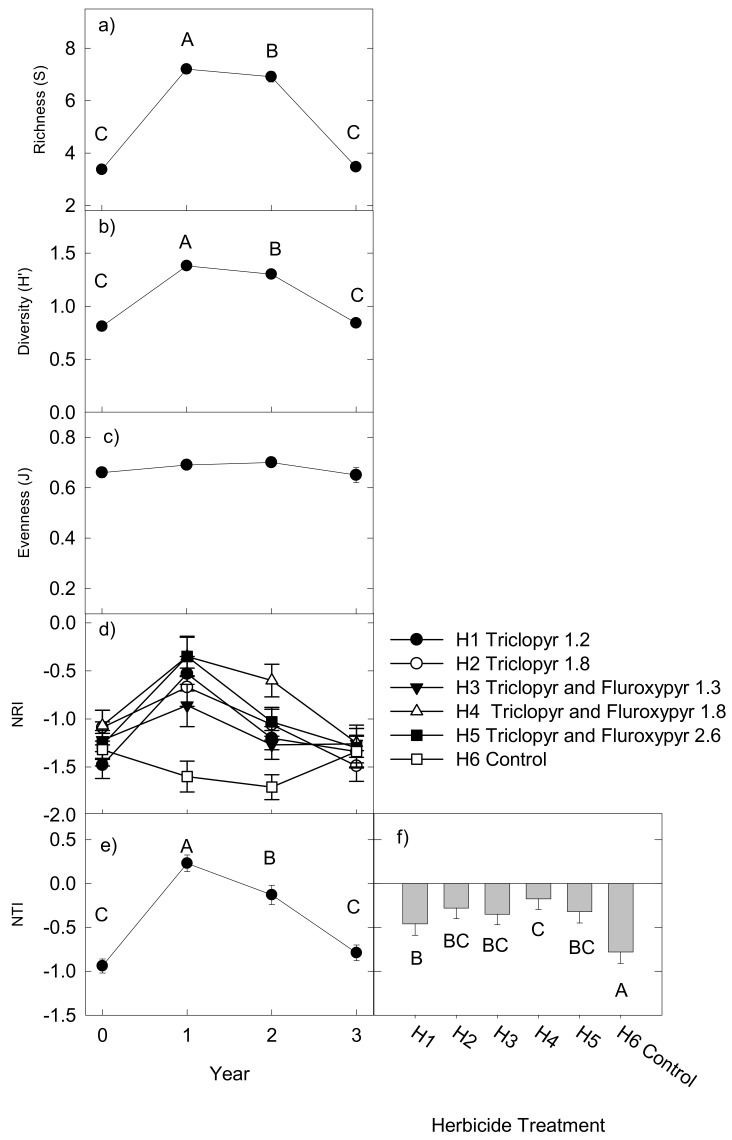
Measures of diversity over four years across three sites (significance shown in Table 1). Mean values sharing the same letter are not significantly different (*p* < 0.05). An interaction between year and herbicide treatment only occurred for NRI (net relatedness index; (**d**) herbicide treatments (H1–H5, H6 = control per Table 3). An herbicide main effect only occurred for NTI (nearest taxon index; (**f**)). Panels (**a**–**c**,**e**) show mean values across herbicide treatments per year (solid circle) ± standard error bar.

Regardless of metric, taxonomic diversity was consistently affected by year (Table 1, Figure 2a–c). Species richness and Shannon’s H’ increased, approximately doubling in year 1 compared with year 0. Evenness (J) showed no significant variation with time. Taxonomic metrics were unaffected by herbicide treatments. The seed addition treatment led to an overall decrease in Shannon’s H’ (seeded plots H’ = 1.02 ± 0.03, unseeded plots H’ = 1.16 ± 0.03). 

Phylogenetic diversity was affected by the herbicide treatments. In years 1 and 2 following herbicide application in year 0, NRI (net relatedness index) decreased in the untreated controls (H6), indicating an increase in phylogenetic over-dispersion. By contrast, all herbicide treatments led to an increase in NRI in years 1 and 2 indicating an increase in clustering. Regardless of time or treatment, the phylogenetic diversity of the vegetation in the sample plots was either not different to a null, random model (NRI: 337 of 515 plots = 65.4%, NTI (nearest taxon index): 416 of 515 plots = 80.8%) or was over-dispersed (i.e., *p* > 0.95, NRI 175 plots = 34.0%, NTI 80 plots = 15.5%). A few plots exhibited clustered phylogenetic diversity (i.e., *p* < 0.05, NRI three plots < 1%, NTI 19 plots = 3.6%).

### 2.4. Effects on Functional Groups

The effects of treatments and year on species richness and cover of functional groups was similar to the effects on taxonomic diversity. At all sites, year had the most pervasive effect with species richness and cover increasing in year 1 and decreasing thereafter to return to year zero values by year 3 (Tables S2 and S3, Figures S1 and S3). Herbicide treatment effects on species richness of functional groups were limited to interactions with the seeding treatment on the number of exotic species (Figure S3) and the cover of all species, exotics, and herbaceous species. In the case of the number of exotic species, the herbicide treatment led to an increase in the number of exotic species compared with untreated controls for treatments H3 and H4 in the seeded plots and in H1, H2, and H3 in the unseeded plots (Figure S2). The herbicide treatments (H1–H5) decreased the total cover compared with untreated controls (H6) in year 2, and decreased the cover of herbaceous species in years 1 and 2. The three-way interaction between herbicide treatment, seeding treatment, and year on the cover of herbaceous species arose because the plots without supplemental seeding had higher cover in the untreated herbicide control plots in year 2 (Figure S3e,j). 

The seeded treatment led to a decrease in the number of herb and Asteraceae species (herbs in seeded plots 3.49 ± 0.12 species per m^2^ and in unseeded plots 3.88 ± 0.11 species per m^2^, Asteraceae species in seeded plots 1.12 ± 0.06 species per m^2^ and in unseeded plots 1.36 ± 0.06 species per m^2^, *p* < 0.05 in both cases; Table S2). The total cover and cover of native species and herbs was decreased in response to seed addition in years 2 and 3 (Figure S3).

### 2.5. Species Composition

Permutational multivariate analysis of variance (PERMANOVA) indicated significant main effects of the herbicide treatment on species composition, and an interaction between year and seed treatment (Table 2). Pairwise multivariate tests indicated that species composition among plots subject to no herbicide (treatment H6, control) was significantly different to plots subject to each of the herbicide treatments (H1 – H5) (*p* < 0.05 in all cases). The year by seed treatment interaction occurred because of significant differences in species composition between seed treatments in years 0 and 1 (*p* < 0.05), but not in years 2 and 3 (*p* > 0.05).

The significant difference between the control treatment and herbicide treatments identified with PERMANOVA was in part due to differences in multivariate dispersion (i.e., permutation test for homogeneity of dispersion (F = 20.12 at 5 degrees of freedom, *p* < 0.0001) with Bray–Curtis distance among herbicide treatment plots (distance to group centroid = 0.41) being significantly smaller than those among the herbicide control plots (mean distance to group centroids = 0.53 ± 0.01, *p* < 0.5 for all comparisons). The year by seed treatment interaction was also reflected in differences in homogeneity of dispersion (F = 24.58 at 5 degrees of freedom, *p* < 0.0001) but differences in homogeneity of dispersion did not occur within a year (*p* > 0.05 for all within-year comparisons). 

## 3. Discussion

Our project had two related goals: first, to assess the short-term efficacy of herbicides to reduce the abundance of the invasive *Lespedeza cuneata*, and second, to assess the extent of any collateral damage to the native plant community. 

The three seasons of data collection after treatments were imposed in this field experiment allowed us to address the first goal and observe the longevity and persistence of *Lespedeza cuneata* control through herbicide application. As expected, *L. cuneata* was best controlled in the season following herbicide application and for one or two seasons thereafter. Herbicide treatments based upon the synthetic auxin triclopyr as the active ingredient have been similarly observed to be most effective for one or two growing seasons after treatment [19]. Triclopyr breaks down rapidly in the environment with a soil half-life of 10–46 days and so persistent control effects are not expected [20]. There was little difference in response of *L. cuneata* among the herbicide treatments indicating that the higher concentrations of triclopyr beyond 1.17 L/ha and the inclusion of the synthetic auxin fluroxypyr treatments may be unnecessary. A similar result contrasting various herbicides including triclopyr and fluroxypyr on the control of *L. cuneata* was observed in native Kentucky, USA grasslands [21]. Regrowth and reestablishment of *L. cuneata* occurred in our restorations by three years. Reestablishment of *L. cuneata* after herbicide treatment is likely due to incomplete mortality of the root system allowing resprouting and seedling establishment from adjacent untreated plants, or both. *Lespedeza cuneata* similarly resprouts and reseeds readily following other disturbances including mowing or fire treatments [22,23].

A second goal of our research was to assess the effects of collateral damage to the native plant community in response to herbicide treatments to control the invasive *L. cuneata*. Collateral damage can be a management dilemma in invasive species control and a challenge in grassland restorations infested with invasives [24,25]. Land managers and restorationists must attempt to limit collateral damage while controlling invasives. For example, managers of a Texas short-grass prairie found that collateral damage from burn treatments imposed to control an invasive C4 grass could be minimized if implemented in a drought year [26]. Our study was also established in a drought year and showed minimal collateral damage effects of the herbicide treatments on the species co-occurring in the community with *L. cuneata.*

The seed addition treatment had only a modest effect on the restorations that were unrelated to the herbicide treatments. Seed additions are undertaken in restorations in an attempt to overcome seed limitation from the local seed pool [27] Adding a “restoration mix” at the start of the growing season following herbicide treatments to control the dominant invasive in our system was undertaken to take advantage of a potential recruitment window. The response to these types of seed addition may take several years to show apparent affects; for example, in one study in a restored grassland it took six years post herbicide treatment [28]. In our study, the seed addition treatment was related to community composition with a decrease in diversity, number of herbaceous and Asteraceae species, and a decrease in total cover and cover of native and herbaceous species, although the decrease in cover was only apparent in the last two years. The single sowing of the seed addition treatment likely limited its chance of success, as although two species not recorded in the plots prior to seeding established from the sown mix, six of what would have been new species did not establish. These results indicate that responses to seed addition treatments may be subtle, even idiosyncratic, and affect particular functional groups. The composition of the original seed mix and of supplemental seed additions used in restorations can affect phylogenetic and functional diversity of the assembling community and is an important consideration in restoration practice [17,29].

Inclusion of phylogenetic diversity in the response metrics in this study showed that aspects of community response to invasive species can control and drive ecosystem function in restorations [17,30]. Phylogenetic diversity can be a consequence of community assembly mechanisms in grassland restorations [31] and is not always congruent with patterns of taxonomic diversity [32]. Such insights from patterns of phylogenetic diversity into mechanisms can be relevant for assessing the impacts of invasive species [33]. In our study, phylogenetic diversity was over-dispersed and related to species composition consistent with mechanisms of competitive exclusion operating in these early successional restorations [34,35]. Phylogenetic diversity was decreased by herbicide treatment (i.e., higher NRI and NTI values) as the plant communities became more phylogenetically clustered in response to herbicide application compared with untreated plots (i.e., certain clades were more or less affected by herbicides than others), which remained over-dispersed. Phylogenetic clustering suggests that there was an increase in abundance of evolutionarily related species with phylogenetically conserved functional traits following control of the abundant *L. cuneata.* Such a pattern is consistent with either environmental filtering or short-term competitive release of subordinate species according to a hierarchical competitive ranking [34,36]. The responses we observed suggest that control of *L. cuneata* allowed competitive release of native species through enhanced growth (higher canopy cover) of a more phylogenetically clustered (at the scale of individual plots) but compositionally variable (among plots) suite of species. The response of the plant community suggests that control of the dominant *L. cuneata* should be continued for a longer period of time through repeated application of herbicides. By contrast, a study of an experimental grassland observed increased phylogenetic diversity in response to broad-spectrum glyphosate herbicide application suggesting that the treatment allowed an increase in abundance of several somewhat unrelated species [37]. 

While herbicides affected *L. cuneata* and the plant community, a strong trend in our data was a temporal effect with most measures of taxonomic and phylogenetic diversity increasing in the year following establishment and subsequently declining in the second or third year. This strong temporal effect may be the response of the community to a drought in the year of establishment of the experiment (i.e., 2012: Table S5) with the communities “rebounding” in the following cooler but moister years regardless of herbicide treatment or seed addition. Droughts can lead to increases in exotic species [38], and may have contributed to the increase in exotic species richness we observed in the second and third years of the experiment (Figure S1b). These restorations exhibited qualities of ecological stability [39] as they returned to their original state following disturbance from drought and herbicide.

## 4. Materials and Methods 

### 4.1. Study Species 

*Lespedeza cuneata* (Dum. Cours.) G. Don is a perennial herbaceous, nitrogen-fixing legume introduced to the United States in 1896 from Asia as forage, and for soil erosion control and reclamation [40,41]. Because of high leaf tannin levels ranging up 58.3 mg/g [42,43], *L. cuneata* is not favored by grazers [44] and has come to dominate many native grasslands and restorations in the eastern United States [45]. It is highly competitive against native species through shading, allelopathy, affecting plant-soil feedbacks through modification of the soil microbiota, and altering rates of soil nitrogen mineralization and nitrification [23,41,46,47,48,49]. The species is difficult to control, and fire, herbicides, grazing and biocontrol have had limited success. High seed production and the buildup of a large soil seed bank of viable seed can also limit success of control efforts [18].

### 4.2. Sites

An experiment was established in summer 2012 at three grassland restoration sites in Crab Orchard National Wildlife Refuge (CONWR) in southern Illinois. The sites were former agricultural fields that differed in management histories (Table S4, Figure S4) with sites 1 and 3 being actively managed grassland restorations. Site 2 was a failed forest restoration and a “Superfund” site with a requirement for hazardous waste cleanup by the US Environmental Protection Agency in which planted trees had not established in the poor and compacted soil. There was a high density of *Lespedeza cuneata* at all three sites. Climate data for the region (Table S5) indicate that the year of establishment of the experiment (2012) had only 61% of normal total precipitation with slightly higher than normal average temperature and twice as many hot days (maximum temperature ≥32 °C) than usual. 

### 4.3. Experimental Design and Treatments

**Figure 3:**
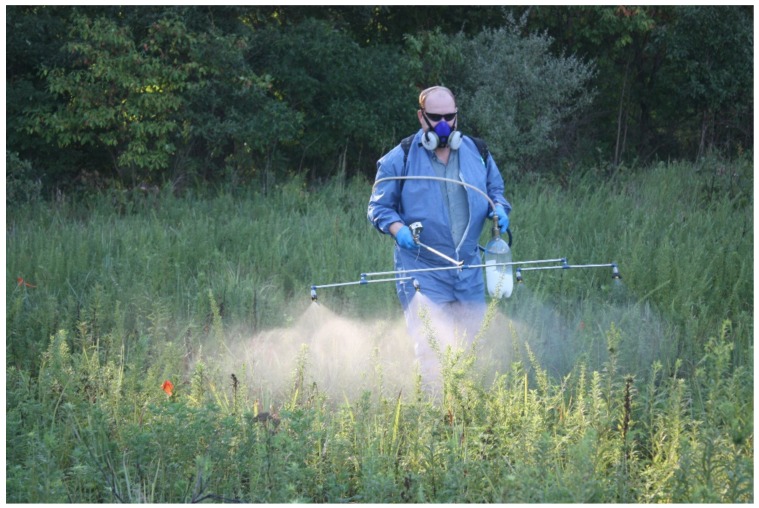
Applying triclopyr and fluroxypyr herbicide to experimental plots dominated by *Lespedeza cuneata*. Photo © David Gibson.

One hundred and forty-four 3 × 6 m plots dominated by *Lespedeza cuneata* were established in summer of 2012 (n = 144; twelve plots in each of four blocks in each of the three sites: Figure S4). The vegetation in each plot was treated on 2 August 2012 with five randomly allocated herbicide treatments of triclopyr and fluroxypyr (Table 3) plus an untreated control. Triclopyr (Garlon 4 Ultra^®^) (3,5,6-Trichloro-2-pyridinyloxyacetic acid) is a systemic herbicide commonly used to control *L. cuneata*. This herbicide internally inhibits growth and targets annual, perennial broadleaf and woody species [50]. Triclopyr in varying rates (Table 3) was applied in combination with fluroxypyr (Pastureguard^®^ HL) ([(4-amino-3,5-dichloro-6-fluoropyridin-2-yl)oxy]acetic acid) using a handheld sprayer (Figure 3). Both are post-emergence synthetic auxins belonging to the pyridine group, and break down readily in the soil due to sunlight and microbial action. Both triclopyr and fluroxypyr are herbicides recommended for use on *L. cuneata* and have been shown to be effective in controlling infestations [50,51]. At the time of application, *L. cuneata* plants were actively growing at the BS (branch-stem) growth stage just prior to flowering when herbicide control with triclopyr and fluroxypyr can be highly effective for up to five growing seasons after treatment [19].

Six months following herbicide application (February 2013), a 14-species Conservation Reserve Program (CRP) seed mix recommended for grassland restorations in the US mid-west (Table S6) was mixed with sand and sown by hand to one half of the herbicide treated areas (seed treatment A). Prior to sowing, the seeds were stratified through refrigeration, with samples germinated in a greenhouse to establish viability and facilitate seedling identification. Immediately before sowing of the seed, plots were hand raked to remove *L. cuneata* litter and to allow seeds contact with the soil. The seed mix was hand broadcast at 300 seeds per m^2^ [52]. 

### 4.4. Data Collection

Density of *L. cuneata* stems (>15 cm in height) was assessed in August 2012 (beginning prior to herbicide treatment and ending 5 days post treatment), as well as in June and August 2013, in June and July 2014, and in August 2015 in 1 m^2^ quadrats centered in each plot. Canopy cover of all vascular species was estimated according to the modified Daubenmire scale [53] in these plots at the same time that density counts of *L. cuneata* stems were made. Maximum cover of each vascular plant species derived from mid-points of the Daubenmire cover classes from the two surveys in 2013 and 2014, and the single surveys in 2012 and 2015, were retained for analyses. 

### 4.5. Statistical Analyses

To improve generality of our results, analyses were conducted across all sites with “site” included as a random factor. All reported values are mean ± standard error of the mean.

The effect of herbicide treatment and seed addition on separate analyses of dependent variables (*L. cuneata* stem density, cover and species richness of functional groups (all species, native species, exotic species, herbaceous (non-grass) species) and dominant families (Poaceae, Fabaceae (not including *L. cuneata*), Asteraceae), Shannon’s diversity, evenness, and phylogenetic diversity) at each site was tested using a repeated measures mixed model in SAS 9.4 [54]. Sites and blocks within sites were included as random effects in each model with year as the repeated measure, and subplot as the experimental unit. Significant differences among means following significant treatment or treatment interaction effects were tested with least squares pairwise tests. The Type I error rate for acceptance of significance was set at α = 0.05.

#### 4.5.1. Phylogenetic Analysis and Tree Construction

A phylogenetic tree of all 75 species across all sites was constructed based on *matK* nucleotide sequences downloaded from GenBank (https://www.ncbi.nlm.nih.gov/genbank/) (Table S1). Of the 75 species, 62 had a *matK* gene represented in GenBank, and for the additional 13 species we used sequences of a congeneric relative. *Nymphaea alba* was used to represent an early diverging Angiosperm lineage as an outgroup and to root the tree. Sequences were aligned using ClustalW and MUSCLE in BioEdit v7.0.5 (http://www.mbio.ncsu.edu/bioedit/bioedit.html) with some manual alignment. Maximum likelihood models were tested in MEGA 7.0 (http://www.megasoftware.net/) with a general time reversible model selected on the basis of having the lowest AIC score (8178.07) among 24 competing nucleotide substitution models and was used to construct a maximum likelihood phylogenetic tree (Figure S5). Alternative phylogenetic trees were not improved when constructed based upon concatenated alignments including *rbcl* and *ITS1* sequences. The phylogenetic tree of our species had no polytomies and was checked against a pruned version of Qian and Jin’s [55] update of Zanne et al.’s [56] megaphlogeny of 31,383 plants. The resulting phylogenetic tree for the species at our sites was imported as a Newick file into R version 3.4.4 [57] where the *picante* package was used to prune the trees to species represented at each of the three sites and calculate abundance-weighted phylogenetic diversity (NTI: nearest taxon index and NRI: net relatedness index) indices for each subplot. NTI and NRI are standardized effect sizes of phylogenetic diversity [58,59]. Positive values of NTI and NRI indicate phylogenetic clustering whereas negative values indicate phylogenetic over-dispersion. The effects of experimental treatments (herbicide and seed addition treatments) on NTI and NRI values were tested using the repeated measures mixed model analysis in SAS with site and blocks within sites as random effects described above for species diversity and cover of functional groups. Post-hoc least squares pairwise tests on significant main effects were calculated.

#### 4.5.2. Species Composition

Differences in species composition (canopy cover) among plots across sites through time (years) in response to herbicide (n = 6 levels) and seed addition (n = 2 levels) treatments and their interactions were assessed through permutational multivariate analysis of variance (PERMANOVA). Post-hoc pairwise comparisons among treatments levels for significant treatments and interactions identified using PERMANOVA were assessed using the pairwise ADONIS package [60]. Multivariate homogeneity of group dispersions (variances) was measured by calculating the average distance of members in herbicide (n = 6 levels) and seed addition (n = 2 levels) treatments to the group centroid respectively, using the BETADISP function in the *vegan* package in R. All multivariate analyses were conducted on Bray–Curtis dissimilarity values among plots in the *vegan* package [61] in R version 3.4.4 [57].

## 5. Conclusions

Herbicide treatment of the invasive exotic was effective, but short term, with the plant communities exhibiting ecological stability following disturbance. Response of the native community to herbicide treatments and supplemental seeding was contingent upon site characteristics and appeared to be more strongly influenced by a recovery from drought rather than competitive release from the invasive. Reduction in abundance of the invasive led to short-term clustered suites of evolutionarily-related native species in plots indicative of more severe environmental filtering on community assembly than biotic filtering in the presence of the invasive.

## Figures and Tables

**Table 1:** F-statistics_degrees of freedom_, and *p*-values (* < 0.05, ** < 0.01, *** < 0.001) from mixed model repeated measures tests on the effects of herbicide and seed addition treatments and their interactions through time (years) on taxonomic (S = richness, H’ = Shannon’s diversity, and E = evenness) and phylogenetic diversity (NRI: net relatedness index, NTI: nearest taxon index) across three prairie restoration sites.

Treatment	S	H	E	NRI	NTI
Year (Y)	338.76_3, 367_***	145.27_3, 364_***	2.00_3, 370_	14.88_3, 361_***	35.90_3, 374_***
Herbicide (H)	0.90_5, 55.2_	0.51_5, 55_	0.23_5, 57_	6.19_5, 49.3_***	4.11_5, 54_**
Seeding (S)	3.84_1, 11.3_	6.98_1, 11.2_*	4.00_1, 10.6_	0.90_1, 11_	1.48_1, 10.8_
Y*H	1.40_15, 374_	0.99_15, 370_	0.66_15, 376_	2.28_15, 365_**	1.23_15, 378_
Y*S	2.09_3, 368_	1.38_3, 367_	4.27_3, 372_	0.86_3, 361_	0.43_3, 375_
H*S	1.75_5, 55.4_	1.72_5, 54.4_	1.09_5, 53.9_	0.75_5, 52.8_	1.99_5, 54.8_
Y*H*S	0.63_15, 375_	0.67_15, 372_	0.75_15, 377_	0.88_15, 365_	0.72_15, 378_

**Table 2:** Results of PERMANOVA tests on the effects of time (year: Y), herbicide treatment (H), seeding treatment (S), and their interactions on Bray–Curtis dissimilarities among plots across all sites. Sites and blocks were included in the analysis as separate main effects without interactions.

Factor				
	df	F	R^2^	P
Sites	1	49.59	0.06	0.001
Blocks	10	20.44	0.25	0.001
Y	1	11.81	0.01	0.001
H	5	4.38	0.03	0.001
S	1	1.94	0.01	0.102
Y*H	5	0.25	<0.01	1.000
Y*S	1	2.88	<0.01	0.004
H*S	5	1.21	<0.01	0.104
Y*H*S	5	0.63	<0.01	0.071
Residuals	504		0.62	
total	538		1.00	

**Table 3:** Summary of herbicide treatments by brand name, active ingredient and concentration in liters per hectare for the plots treated in summer 2012.

Treatment	Herbicide Brand Name	Active Ingredient (s)	Rate (L. ha^-1^)
H1	Garlon 4 Ultra^®^	Triclopyr	1.17
H2	Garlon 4 Ultra^®^	Triclopyr	1.76
H3	Pastureguard^®^ HL	Triclopyr and Fluroxypyr	1.29
H4	Pastureguard^®^ HL	Triclopyr and Fluroxypyr	1.76
H5	Pastureguard^®^ HL	Triclopyr and Fluroxypyr	2.57
H6	Control	N/A	N/A

## Supplementary Materials

The following are available online at https://www.mdpi.com/2223-7747/8/10/426/s1, **Figure S1:** Number of species (number per m^2^) by functional group over four years across three sites (significance shown in Table S2). Mean values sharing the same letter are not significantly different (*p* < 0.05). **Figure S2:** Number of exotic species (number per m^2^) in response to significant herbicide by seed treatment interaction (significance shown in Table S2). Mean values sharing the same letter are not significantly different (*p* < 0.05). Herbicide treatments (H1–H5, H6 = control per Table 3). **Figure S3:** Cover of species (%) by functional group over four years across three sites (significance shown in Table S3). Mean values sharing the same letter are not significantly different (*p* < 0.05). The significant interactions between year and seed treatment effects are shown for total cover, native species, and herbaceous species (S1 = seeded treatment, S2 = unseeded). There were herbicide treatment interactions with year for total cover, and a three-way interaction effect between year, seed treatment, and herbicide treatment on the cover of herbaceous species (for clarity the two two-way interactions between year and seed treatment and year and herbicide treatment are shown). Herbicide treatments (H1–H5, H6 = control per Table 3). **Figure S4:** Schematic maps of each site showing block layout and orientation within these sites. A and B refer to unseeded (A) and seeded strips of plots within each block (see Table S4). T1–T6 refers to herbicide treatments (H1-H6, see Table 3). Triangles correspond to the asterisks on these maps for block orientation. GPS coordinates for each block are: Site 1: Block 1-N 37 43.134 W 089 01.597, Block 2-N 37 43.141 W 089 01.592, Block 3-N 37 43.154 W 089 01.686, Block 4-N 37 43.143 W 089 01.696; Site 2: Block 5-N 37 43.461 W 089 02.394, Block 6-N 37 43.471 W 089 02.394, Block 7-N 37 43.570 W 089 02.353, Block 8-N 37 43.515 W 089 02.292; Site 3: Block 9-N 37 41.536 W 089 03.146, Block 10-N 37 41.554 W 089 03.147, Block 11-N 37 41.563 W 089 03.161, Block 12-N 37 41.573 W 089 03.154. **Figure S5** Phylogenetic tree of all species encountered across the three sites from 2013 to 2016. Species codes as in Table S1. *Nymphaea alba* (Nyal) was included in the phylogeny as an outgroup. **Table S1.** Species names and codes used in Figure S5 (BioEdit code), associated plant families, *matK* GenBank accession number, and names of congeners used instead of species where GenBank accessions were not available. **Table S2.** F-statistics,_degrees of freedom_, and *p*-values (* < 0.05, ** < 0.01, *** < 0.001) from mixed model repeated measures tests on the effects of herbicide (Table 3) and seed addition treatments (Table S6) and their interactions through time (years) on numbers of species across three prairie restoration sites. The life form ‘Legumes’ include species in the Fabaceae but not *Lespedeza cuneata*. ‘Asters’ includes species in the Asteraceae. Species counts were log transformed to improve normality where indicated ^ᵻ^. **Table S3.** F-statistics,_degrees of freedom_, and *p*-values (* < 0.05, ** < 0.01, *** < 0.001) from mixed model repeated measures tests on the effects of herbicide (Table 3) and seed addition treatments (Table S6) and their interactions through time (years) on cover of species functional groups across three prairie restoration sites. The life form ‘Legumes’ include species in the Fabaceae but not *Lespedeza cuneata*. ‘Asters’ includes species in the Asteraceae. Species counts were log transformed to improve normality where indicated ^ᵻ^. **Table S4**: Summary of each study site and management in CONWR (Personal Communication Refuge Manager Mike Brown, 2007). **Table S5.** Summary climate data for southern Illinois regional airport, Williamson County (Source www.wunderground.com). Historical averages 1973–2014 from Carbondale Sewage Plant, Jackson County (Source www.noaa.gov). **Table S6**: Summary of CRP seed mix (CP-33: tall grass mix) applied to plots. Mix was applied to plots at an approximate density of 300 seeds per m^2^.
