# Supplementary material for: Do No Harm: Efficacy of a Single Herbicide Application to Control an Invasive Shrub While Minimizing Collateral Damage to Native Species
Source: Plants (Basel). 2019 Oct 18;8(10):426. doi: 10.3390/plants8100426 (PMC6843660; doi:10.3390/plants8100426)

Do no harm: Efficacy of a single herbicide application to control an invasive shrub while minimizing collateral damage to native species

David J. Gibson, Lindsay A. Shupert, Xian Liu

Supplemental Material

Table S1. Species names and codes used in Figure S5 (Bioedit code), associated plant families, matK GenBank accession number, and names of congeners used instead of species where GenBank accessions were not available.

Bioedit code	Datafile code	Species name	Plant Family	matk GenBank number	Congener used when necessary
Acrh	ACCRH	*Acalypha rhomboidea*	Euphorbiaceae	KP643092.1	
Acmi	ACHMI	*Achillea millefolium*	Asteraceae	KP210356.1	
Alvi	ALLVI	*Allium vineale*	Alliaceae	HM850504.1	
Amar	AMBEL	*Ambrosia artemisifolia*	Asteraceae	HQ593164.1	
Amal	AMPAL	*Ampelamus albidus*	Asclepiadaceae	JQ586765.1	*Cynanchum racemosum*
Ange	ANOGE	*Andropogon gerardii*	Poaceae	AF144577.1	
Anne	ANXNE	*Antennaria neglect*	Asteraceae	HQ593172.1	
Apca	APCCA	*Apocynum cannabinum*	Apocynaceae	HQ384552.1	
Assy	ASCSY	*Asclepias syriaca*	Asclepiadaceae	DQ660501.1	
Brra	BRORA	*Bromus racemosus*	Poaceae	JN895847.1	
Cara	CMIRA	*Campsis radicans*	Bignoniaceae	GQ434139.1	
Cabi	CAREX	*Carex sp.*	Cyperaceae	KU496568.1	
Chfa	CASFA	*Chamaecrista fasciculata*	Caesalpiniaceae	GQ248093.1	
Cidi	CIRDI	*Cirsium discolor*	Asteraceae	KP643137.1	
Coar	CONAR	*Convolvulus arvensis*	Convolvulaceae	KT176616.1	
Eric	ERICA	*Conyza canadensis*	Asteraceae	KJ204460.1	
Cola	CRLLA	*Coreopsis lanceolata*	Asteraceae	AY551495.1	
Cupe	CUSPE	*Cuscuta pentagona*	Cuscutaceae	EU330285.1	*C. reflexa*
Depa	DESMO	*Desmodium sp.*	Fabaceae	KJ772717.1	
Diar	DINAR	*Dianthus armeria*	Caryophyllaceae	KP210382.1	
Diol	PANSC	*Dichanthelium oligosanthes*	Poaceae	KJ740971.1	
Elvi	ELYVIR	*Elymus virginicus*	Poaceae	KJ592936.1	
Eran	ERIAN	*Erigeron annuus*	Asteraceae	HQ593285.1	
Euco	EPHCO	*Euphorbia corollata*	Euphorbiaceae	KP642990.1	
Fear	FESAR	*Festuca arundinaceum*	Poaceae	HQ593347.1	

Gaap	GALAP	*Galium sp.*	Rubiaceae	KJ772810.1	*Galium tinctorium*
Geca	GERCA	*Geranium carolinianum*	Geraniaceae	EU922172.1	
Hopu	HORPU	*Hordeum pusillum*	Poaceae	AB078133.1	
Ipla	IPOLA	*Ipomoea lacunosa*	Convolvulaceae	AY491651.1	*Ipomoea mauritiana*
Juox	JUNCUS	*Juncus sp*	Juncaceae	HQ593335.1	
Lase	LACSE	*Lactuca serriola/scariola*	Asteraceae	AJ633237.1	
Leca	LESCP	*Lespedeza capitata*	Fabaceae	KJ772888.1	
Lecu	LESCU	*Lespedeza cuneata*	Fabaceae	KP094023.1	
Lias	LTSAS	*Liatris aspera*	Asteraceae	HQ416287.1	*Liatris spicata*
Loja	LONJA	*Lonicera japonica*	Caprifoliaceae	HM228456.1	
Melu	MEDLU	*Medicago lupulina*	Fabaceae	KJ204508.1	
Meal	MEUAL	*Melilotus albus*	Fabaceae	HQ593364.1	
Meof	MEUOF	*Melilotus officinalis*	Fabaceae	KP987698.1	
Nyal		*Nymphaea alba*	Nymphaeaceae	JN894938.1	(Outgroup species)
Oxst	OXAST	*Oxalis stricta*	Oxalidaceae	AY935936.1	
Pavi	PANVI	*Panicum virgatum*	Poaceae	EU434294.1	
Pahy	PARIT	*Parthenium integrifolium*	Asteraceae	HM989799.1	*Parthenium hysterophorus*
Pedi	PEEDI	*Penstemon digitalis*	Scrophulariaceae	KT176610.1	*Penstemon angustifolius*
Phar	TYPAR	*Phalaris arundinacea*	Poaceae	HQ593381.1	
Phpr	PHLPR	*Phleum pratense*	Poaceae	HQ593382	
Phsu	SOLPS	*Physalis subglabrata*	Solanaceae	EU128756.1	*Physalis subglabrata*
Plar	PLAAR	*Plantago aristata*	Plantaginaceae	KJ773013.1	
Plla	PLALA	*Plantago lanceolata*	Plantaginaceae	KJ204519.1	
Poco	POACO	*Poa compressa*	Poaceae	KJ529347.1	
Popr	POAPR	*Poa pratensis*	Poaceae	HQ593389	
Posa	PYGSA	*Polygala sanguina*	Polygalaceae	KJ773028.1	*Polygala polygama*
Pyte	PJHSS	*Pycnanthemum tenuifolium*	Lamiaceae	KJ773052.1	*Pycnanthemum floridanum*
Qusp	QUERC	*Quercus sp.*	Fagaceae	KJ593084.1	*Quercus velutina*
Rhco	RHUCO	*Rhus copallina*	Anacardiaceae	AY594485.1	

Romu	ROSMU	*Rosa multiflora*	Rosaceae	AB011991.1	
Ruar	RUBAG	*Rubus argutus*	Rosaceae	KJ773096.1	
Ruhi	RUDHI	*Rudbeckia hirta*	Asteraceae	HQ593421.1	
Rucr	RUMCR	*Rumex crispus*	Polygonaceae	HQ593423.1	
Ansc	ANOSC	*Schizachyrium scoparium*	Poaceae	FR832830.1	
Czva	CZRVA	*Securigera varia*	Fabaceae	HM049547.1	
Sefa	SETFR	*Setaria faberii*	Poaceae	KF163775.1	
Segl	SETLU	*Setaria glauca*	Poaceae	FR667667.1	*Setaria italica*
Siin	SIPIN	*Silphium integrifolium*	Asteraceae	KT176592.1	
Sipe	SIPTE	*Silphium terebinthinaceum*	Asteraceae	AY215859.1	*Silphium perfoliatum*
Smga	SMIGL	*Smilax glauca*	Smilacaceae	KJ773155.1	
Slca	SOLCA	*Solanum carolinense*	Solanaceae	KP642773.1	
Soca	SOOCA	*Solidago Canadensis*	Asteraceae	EU749415.1	
Eugr	SOOGR	*Solidago graminfolia*	Asteraceae	KJ592944.1	
Sonu	SOSNU	*Sorghastrum nutans*	Poaceae	KC123429.1	
Soha	SORHP	*Sorghum halepense*	Poaceae	KF163824.1	
Toja	TOIJA	*Torilis japonica*	Apiaceae	JN895299.1	
Trfl	TRIFL	*Tridens flavus*	Poaceae	HM352810.1	
Trca	TRFCA	*Trifolium campestre*	Fabaceae	JN894254.1	
Trre	TRFRE	*Trifolium repens*	Fabaceae	JN894453.1	
Trpe	TJDBI	*Triodanis biflora*	Campanulaceae	EU713256.1	

Table S2. F-statistics,$_{\text{degrees of freedom}}$, and P values (* < 0.05, ** < 0.01, *** < 0.001) from mixed model repeated measures tests on the effects of herbicide and seed addition treatments (Tables 4 & S7) and their interactions through time (years) on numbers of species across three prairie restoration sites. The life form 'Legumes' include species in the Fabaceae but not *Lespedeza cuneata*. 'Asters' includes species in the Asteraceae. Species counts were log transformed to improve normality where indicated ‡.

Treatments	Natives	Exotics ‡	Grasses	Herbs ‡	Legumes	Asteraceae	Seeded
Year (Y)	**$187.76_{3,353}^{***}$**	**$148.60_{3,372}^{***}$**	**$188.63_{3,364}^{***}$**	**$161.25_{3,368}^{***}$**	**$77.25_{3,365}^{***}$**	**$50.28_{3,3.62}^{***}$**	**$14.27_{3,362}^{***}$**
Herbicide (H)	$1.90_{5,53.8}$	$1.77_{5,52.9}$	$1.19_{5,52.8}$	$0.31_{5,56.6}$	$2.15_{5,53.7}$	$0.30_{5,52.4}$	$0.96_{5,54.8}$
Seeding (S)	$1.77_{1,1.64}$	$2.96_{1,11.2}$	$0.10_{1,11.2}$	**$5.84_{1,11.4}^{*}$**	$1.50_{1,11.5}$	**$8.08_{1,11.6}^{*}$**	$0.93_{1,11.5}$
Y*H	$1.37_{15,359}$	$1.09_{15,379}$	$1.07_{15,370}$	$1.20_{15,373}$	$1.18_{15,371}$	$1.40_{15,368}$	$0.87_{15,369}$
Y*S	$1.23_{3,355}$	$0.91_{3,374}$	$0.74_{3,367}$	$1.20_{3,370}$	$1.75_{3,368}$	$1.15_{3,366}$	$1.02_{3,366}$
H*S	$0.85_{5,51.9}$	**$3.19_{5,57}^{*}$**	$1.51_{5,53.3}$	$1.55_{5,55.9}$	$0.41_{5,54.6}$	$0.93_{5,53.2}$	$1.78_{5,54.3}$
Y*H*S	$0.72_{15,360}$	$0.57_{15,379}$	$1.92_{15,371}$	$0.91_{15,375}$	$0.97_{15,371}$	$1.12_{15,369}$	$1.26_{15,370}$

Table S3. F-statistics,$_{\text{degrees of freedom}}$, and P values (* < 0.05, ** < 0.01, *** < 0.001) from mixed model repeated measures tests on the effects of herbicide and seed addition treatments (Tables 3 & S6)and their interactions through time (years) on cover of species functional groups across three prairie restoration sites. The life form 'Legumes' include species in the Fabaceae but not *Lespedeza cuneata*. 'Asters' includes species in the Asteraceae. Species counts were log transformed to improve normality where indicated [Ɨ].

Treatments	All species	Natives	Exotics [Ɨ]	Grasses[Ɨ]	Herbs	Legumes[Ɨ]	Asteraceae[Ɨ]	Seeded[Ɨ]
Year (Y)	$\mathbf{140.43_{3,374}}$***	$\mathbf{82.57_{3,368}}$***	$\mathbf{3.57_{3,368}}$*	$\mathbf{103.19_{3,363}}$***	$\mathbf{35.88_{3,363}}$***	$\mathbf{64.08_{3,369}}$***	$\mathbf{32.40_{3,369}}$***	$\mathbf{11.25_{3,368}}$***
Herbicide (H)	$1.20_{5,55.9}$	$1.49_{5,55.5}$	$2.39_{5,56.6}$*	$1.38_{5,53.6}$	$\mathbf{5.03_{5,54.7}}$*	$2.24_{5,56.4}$	$0.37_{5,57.1}$	$0.96_{5,48.9}$
Seeding (S)	$1.12_{1,11.3}$	$4.67_{1,11}$	$0.40_{1,11.4}$	$0.37_{1,11.1}$	$2.43_{1,1.1}$	$1.65_{1,11.4}$	$4.75_{1,11}$	$1.10_{1,11.6}$
Y*H	$\mathbf{1.99_{15,379}}$*	$1.49_{15,373}$	$\mathbf{1.87_{15,371}}$*	$0.73_{15,370}$	$\mathbf{2.69_{15,369}}$**	$0.61_{15,375}$	$1.01_{15,375}$	$1.18_{15,334}$
Y*S	$\mathbf{3.82_{3,376}}$*	$\mathbf{3.82_{3,369}}$*	$2.49_{3,369}$	$0.75_{3,367}$	$\mathbf{6.11_{3,366}}$**	$1.48_{3,372}$	$0.03_{3,371}$	$0.82_{3,352}$
H*S	$0.49_{5,56}$	$1.26_{5,54.6}$	$1.06_{5,56.3}$	$0.26_{5,52.5}$	$0.46_{15,56.8}$	$0.04_{5,56}$	$1.97_{3,56.9}$	$1.18_{5,52.1}$
Y*H*S	$1.52_{15,379}$	$0.68_{15,374}$	$0.43_{15,374}$	$0.85_{15,371}$	$\mathbf{2.11_{15,369}}$**	$0.89_{15,376}$	$1.17_{15,377}$	$0.93_{15,354}$

Table S4: Summary of each study site and management in CONWR (Personal Communication Refuge Manager Mike Brown, 2007).

Site Name	Size and Location	History and Management
**Site 1: Headquarters Prairie**	**1.** 6.9 ha<br>**2.** Located off of Pigeon Creek Road	**1.** Established 1971<br>**2.** Prescribed burns in 1974, 1977, 1981, 1987, 1989, 1990, 1991, 1994, 1997, 2000, 2002, 2004, 2006, 2008, 2010, 2012 and 2014<br>**3.** Unit was mowed in August 2001 in an attempt to control *Rhus* spp. and sprayed with Plateau herbicide to control *L. cuneata* in 2007<br>**4**. 1600 native prairie plants (rootstock) added in 1997, species included: *Echinacea sp., Eryngium yuccifolium, Silphium integrifolium, S. laciniatum, Liatris spicata, Parthenium integrifolium, Physostegia virginiana*
**Site 2: Area 36**	**1.** Approximately 20 ha<br>**2.** Located off of Pigeon Creek Road, east of Stringtown Road	**1.** Site of former sewage treatment plant (EPA cleanup completed 2006)<br>**2.** Tree plantings were unsuccessful dueo to severe soil compaction<br>**3.** Seed added (date and species unknown)<br>**4.** No record of fire treatment
**Site 3: Bass Pond**	**1.** 12.1 ha<br>**2.** Located off of Wolf Creek Road, east of observation deck	**1.** Established around 1990<br>**2.** Prescribed burns beginning in 1993, and continuing in 1996, 1998, 2001, 2003, 2005, 2007, 2008, 2010, 2012 and 2014<br>**2.** Reseeded with native prairie species (date and species unknown) and treated with Plateau herbicide to control *L. cuneata* in 2007.

Table S5. Summary climate data for southern Illinois regional airport, Williamson County (Source www.wunderground.com). Historical averages 1973-2014 from Carbondale Sewage Plant, Jackson County (Source www.noaa.gov).

	2012	2013	2014	2015	Historical averages
Average high temperature (C)	21.7[1.]	18.3	17.8	19.4	19.3
Average low temperature (C)	8.9	7.2	6.7	8.3	6.8
Average mean temperature (C)	15.0	12.8	12.2	13.8	13.1
Total precipitation (cm)	73.3	106.4	82.5	126.2	116.1

[1.] 2012 had 79 days with a maximum daily temperature equal to or exceeding 90 F (32 C) compared with the historical average of 39 days.

Table S6: Summary of CRP seed mix (CP-33: tall grass mix) applied to plots. Mix was applied to plots at an approximate density of 300 seeds per m^2. * = sown species that did not establish. † = already present in plots prior to seeding.

Species	Common Name	Family	% in Mix
Andropogon gerardii †	Big Blue Stem	Poaceae	15.41
*Bouteloua curtipendula**	Sideoats grama	Poaceae	15.41
Schizachyrium scoparium †	Little Blue Stem	Poaceae	30.83
Sorghastrum nutans †	Indian grass	Poacea	15.41
Chamaecrista fasciculata †	Partridge Pea	Caesalpiniaceae	3.85
*Dalea candidum**	White Prairie Clover	Fabaceae	3.85
*Dalea purprea**	Purple Prairie Clover	Fabaceae	3.85
Desmanthus illinoensis	Illinois Bundle Flower	Fabaceae	3.85
*Echinacea pallida**	Pale Purple Coneflower	Asteraceae	1.93
Lespedeza capitata	Bush Clover	Fabaceae	0.96
Penstemon digitalis †	Foxglove Beardtongue	Scrophulariaceae	0.39
*Ratibida pinnata**	Yellow Coneflower	Asteraceae	1.93
Rudbeckia hirta	Black-eyed Susan	Asteraceae	1.93
*Veronicastrum virginicum**	Culver's root	Scrophulariaceae	0.39

Figure S1 Number of species (number per m^2) by functional group over four years across three sites (significance shown in Table S2). Mean values sharing the same letter are not significantly different ($P < 0.05$).

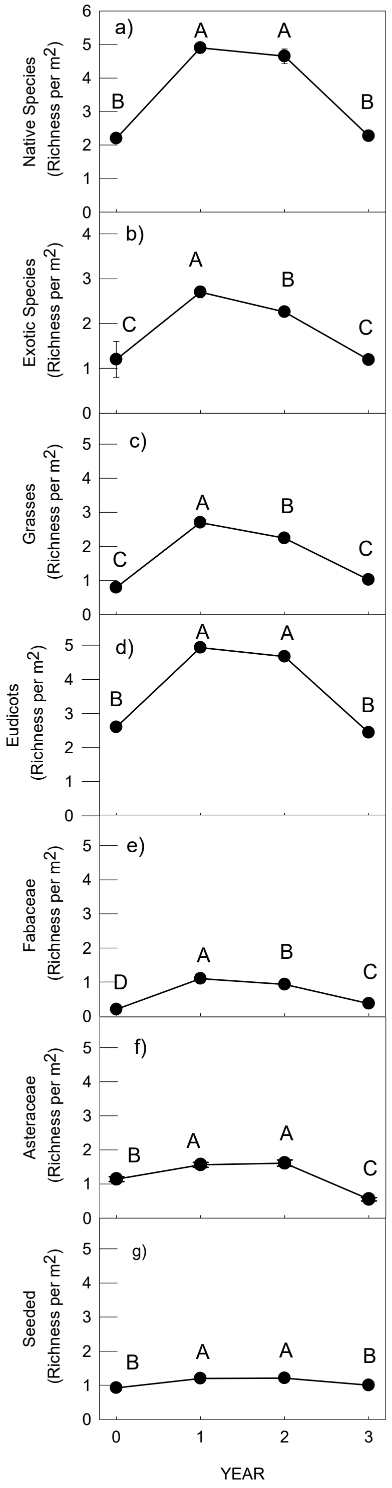

Figure S2 Number of exotic species (number per m^2) in response to significant herbicide by seed treatment interaction (significance shown in Table S2). Mean values sharing the same letter are not significantly different ($P < 0.05$). Herbicide treatments (H1-H5, H6 = control per Table 3).

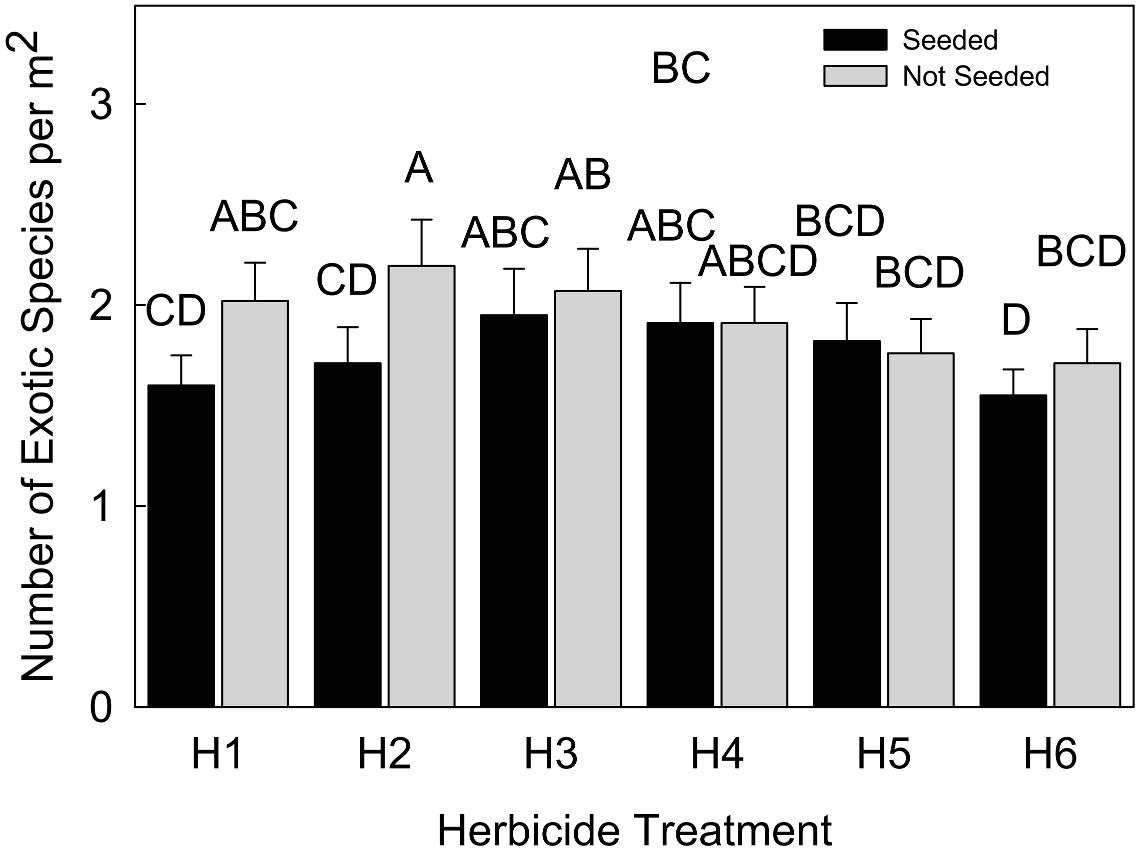

Figure S3 Cover of species (% ± se) by functional group over four years across three sites (significance shown in Table S3). Mean values sharing the same letter are not significantly different ($P < 0.05$). The significant interactions between year and seed treatment effects are shown for total cover, native species, and herbaceous species (S1 = seeded treatment, S2 = unseeded). There were herbicide treatment interactions with year for total cover, and a three-way interaction effect between year, seed treatment, and herbicide treatment on the cover of herbaceous species (for clarity the two two-way interactions between year and seed treatment and year and herbicide treatment are shown). Mean values are shown for herbicide treatments (H1-H5, H6 = control per Table 3) for panels c), i), and j), for seeded treatments for panels a), b), and e), other panels show the mean over all treatments (solid circles).

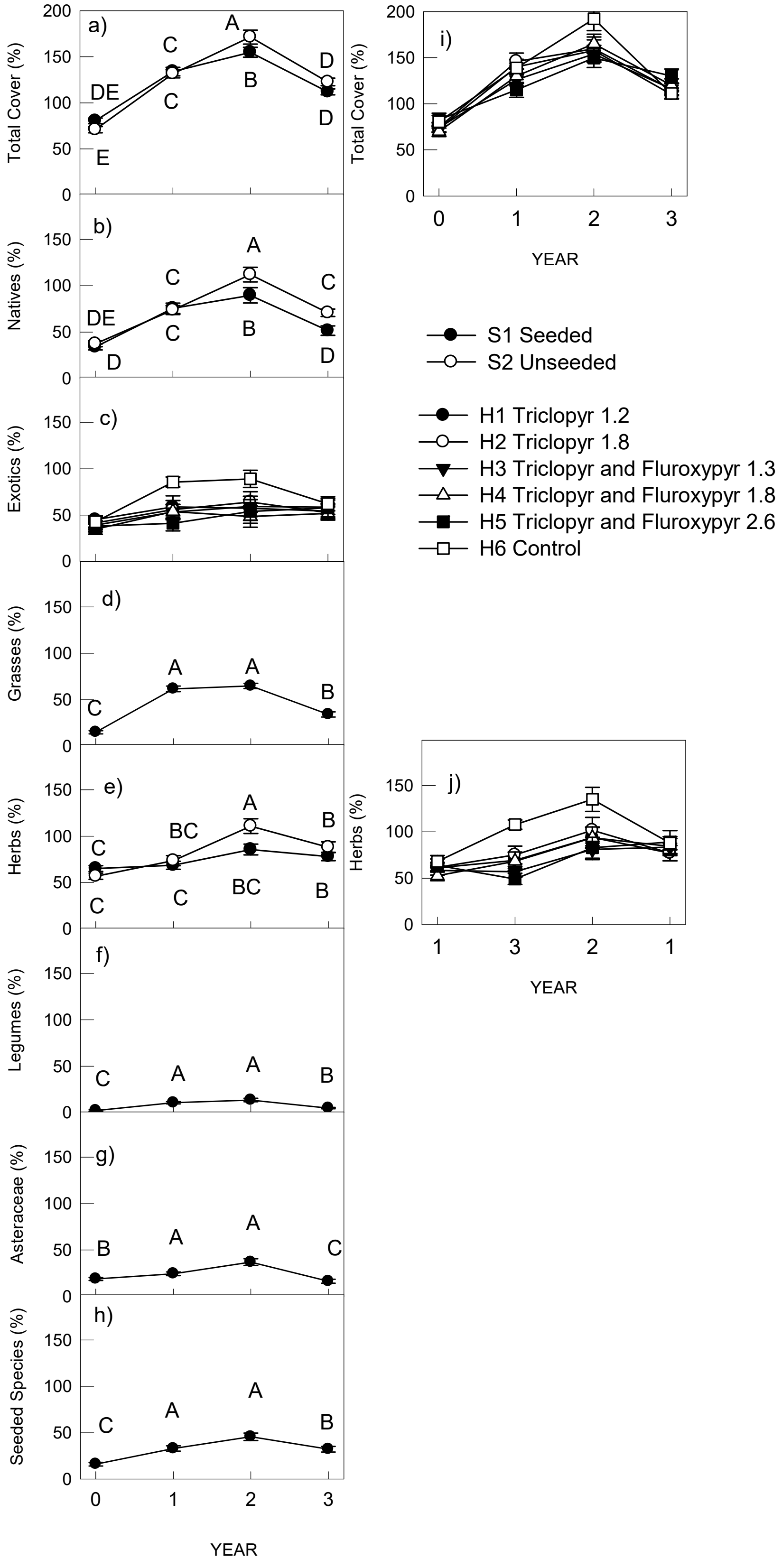
a)
b)
c)
d)
e)
f)
g)
h)
i)
j)
Total Cover (%)
Natives (%)
Exotics (%)
Grasses (%)
Herbs (%)
Legumes (%)
Asteraceae (%)
Seeded Species (%)
YEAR
S1 Seeded
S2 Unseeded
H1 Triclopyr 1.2
H2 Triclopyr 1.8
H3 Triclopyr and Fluroxypyr 1.3
H4 Triclopyr and Fluroxypyr 1.8
H5 Triclopyr and Fluroxypyr 2.6
H6 Control

Figure S4: Schematic maps of each site showing block layout and orientation within these sites. A and B refer to unseeded (A) and seeded strips of plots within each block (see table S7). T1-T6 refers to herbicide treatments (H1-H6, see Table 3). Triangles correspond to the asterisks on these maps for block orientation. GPS coordinates for each block are: Site 1: Block 1- N 37 43.134 W 089 01.597, Block 2-N 37 43.141 W 089 01.592, Block 3- N 37 43.154 W 089 01.686, Block 4- N 37 43.143 W 089 01.696; Site 2: Block 5- N 37 43.461 W 089 02.394, Block 6- N 37 43.471 W 089 02.394, Block 7- N 37 43.570 W 089 02.353, Block 8- N 37 43.515 W 089 02.292; Site 3: Block 9- N 37 41.536 W 089 03.146, Block 10- N 37 41.554 W 089 03.147, Block 11- N 37 41.563 W 089 03.161, Block 12- N 37 41.573 W 089 03.154.

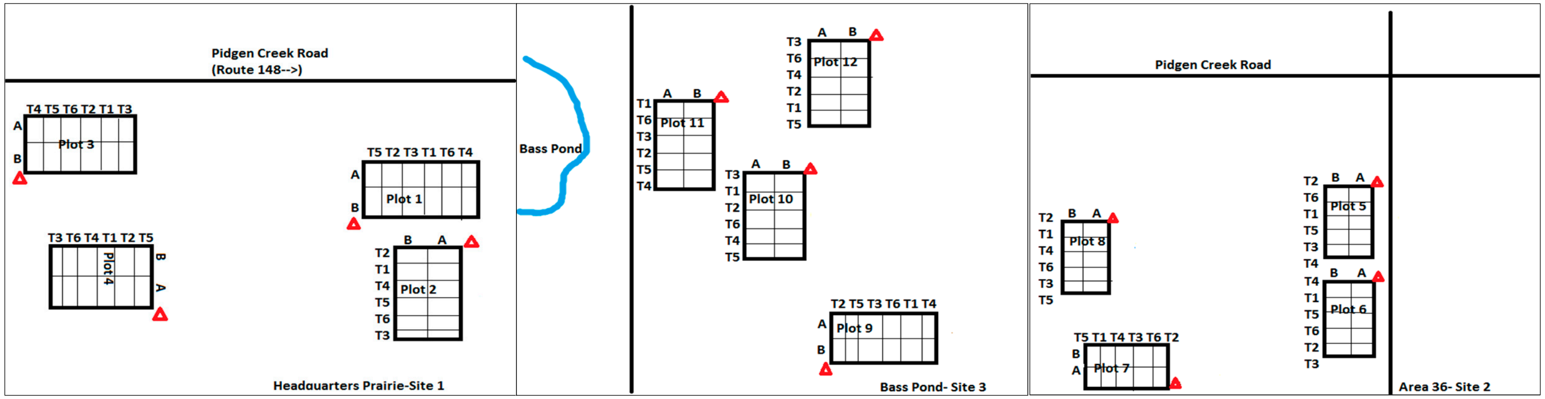

Figure S5 Phylogenetic tree of all species encountered across the three sites from 2013 – 2016. Species codes as in Table S1. *Nymphaea alba* (Nyal) was included in the phylogeny as an outgroup.

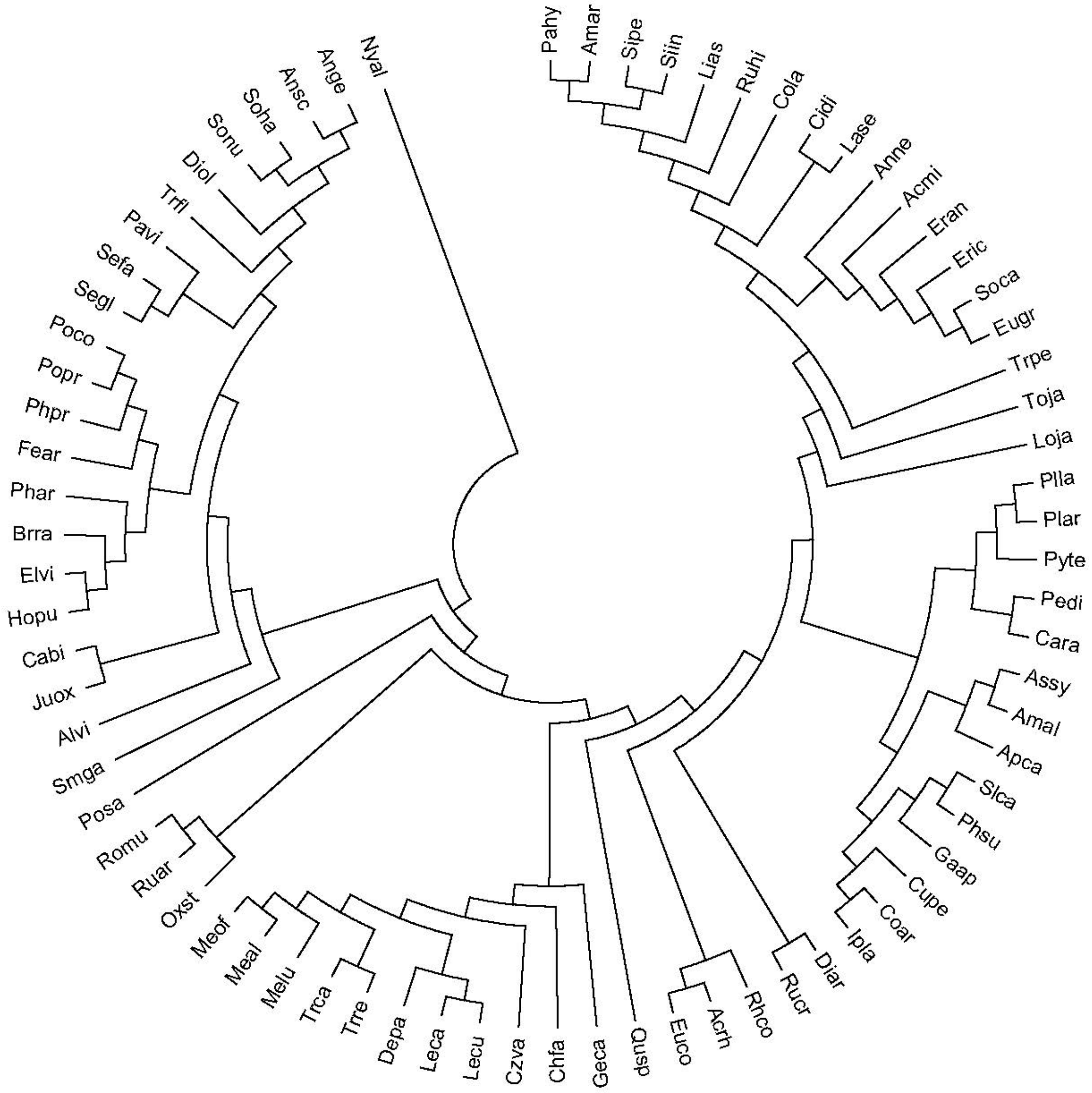

Supplement: Supplementary file 1 [file plants-08-00426-s001.pdf]
